# The effect of hearing loss on a rat model with genetic susceptibility to Alzheimer's disease

**DOI:** 10.1002/alz70855_104043

**Published:** 2025-12-24

**Authors:** Salonee V Patel, Sarah J Myers, Ashley L Schormans, Sophia MH Robinson, Parinita Arora, Shawn N Whitehead, Brian L Allman, Sarah H Hayes

**Affiliations:** ^1^ Western University, London, ON, Canada; ^2^ University of Rochester, Rochester, NY, USA

## Abstract

**Background:**

Dementia affects ∼55 million people worldwide; a staggering statistic which highlights the need to limit its risk factors. Cognitive decline and sensory gating deficits are all hallmark of Alzheimer's disease (AD), and are thought to be mediated in part by several neuropathological changes within the brain including synaptic loss in the cerebral cortex and hippocampus. Importantly, epidemiological studies have identified hearing loss as a major modifiable risk factor for the development of dementia, including AD. However, the mechanisms by which hearing loss interacts with genetic susceptibility to increase the risk of developing AD is poorly understood.

**Methods:**

In the present study, we investigated the effect of hearing loss on cognitive function, sensory gating, and hippocampal synaptic density in a rat model with genetic susceptibility to AD. Fischer 344 (TgAPP) rats represent a prodromal model of AD, as they overexpress pathogenic human amyloid precursor protein, but do not spontaneously develop β‐amyloid plaques. At 15 months of age, TgAPP male rats were noise‐exposed (12 kHz, 110 dB SPL, 3 hours) to generate hearing loss, with sham‐exposed wildtype and TgAPP rats utilized as controls. Cognitive function was assessed using the Morris water maze task, with hippocampal synaptic density quantified through Golgi‐Cox staining. Sensory gating was assessed behaviourally using pre‐pulse inhibition of the acoustic startle reflex and electrophysiologically with an auditory paired pulse paradigm.

**Results:**

Consistent with hearing loss often experienced by humans, our protocol resulted in high‐frequency hearing loss in the rats. As expected, TgAPP rats exhibited impaired reference memory compared to the wildtype rats; however, this was not exacerbated by hearing loss. While behavioural sensory gating remained unchanged, our preliminary electrophysiological results revealed genotype‐specific differences in auditory gating. Lastly, we observed an increase in spine density on basal dendrites of hippocampal CA1 neurons in noise‐exposed animals.

**Conclusion:**

Ultimately, our preclinical data help provide novel insight into the interplay between hearing loss and genetic susceptibility to AD. Collectively, our results demonstrate that while AD‐related impairments were evident in our prodromal model of AD, the degree of noise‐induced hearing loss used in the present study was insufficient to exacerbate these impairments.

